# The Gln32Lys Polymorphism in HSP22 of Zhikong Scallop *Chlamys farreri* Is Associated with Heat Tolerance

**DOI:** 10.1371/journal.pone.0028564

**Published:** 2011-12-05

**Authors:** Chuanyan Yang, Lei Zhang, Lingling Wang, Huan Zhang, Limei Qiu, Vinu S. Siva, Linsheng Song

**Affiliations:** 1 The Key Laboratory of Experimental Marine Biology, Institute of Oceanology, Chinese 7 Academy of Sciences, Qingdao, China; 2 Graduate School, Chinese Academy of Sciences, Beijing, China; New England Biolabs, Inc., United States of America

## Abstract

**Background:**

Heat shock protein 22 is a member of small heat shock proteins with molecular chaperone activity. Though their multiple functions have been well characterized, there is no report about the association between the polymorphisms of HSP22 and heat tolerance.

**Methodology:**

Three single nucleotide polymorphisms were identified in HSP22 from scallop *Chlamys farreri* (CfHSP22), and the +94 C-A locus was found to be nonsynonymous. Three genotypes at locus +94, A/A, A/C and C/C, were revealed by using Bi-PASA PCR analysis, and their frequencies were 19.5%, 27.6% and 52.9% in the heat resistant stock, while 9.3%, 17.4% and 73.3% in the heat susceptible stock, respectively. The frequency differences of the three genotypes were significant (*P*<0.05) between the two stocks. After incubating at 30°C for 84 h, the cumulative mortality of scallops with +94 C/C genotype and +94 A/C genotypes was 95% and 90%, respectively, which was significantly higher (*P*<0.01) than that of scallops with +94 A/A genotype (70%). The molecular chaperone activity of two His-tagged fusion proteins, rCfHSP22Q with +94 C/C genotype and rCfHSP22K with +94 A/A genotype were analyzed by testing the ability of protecting citrate synthase (CS) against thermal inactivation *in vitro*. After incubated with rCfHSP22Q or rCfHSP22K at 38°C for 1 h, the activity of CS lost 50% and 45%, and then recovered to 89% and 95% of the original activity following 1 h restoration at 22°C, respectively, indicating that the mutation from Gln to Lys at this site might have an impact on molecular chaperone activities of CfHSP22.

**Conclusions:**

These results implied that the polymorphism at locus +94 of CfHSP22 was associated with heat tolerance of scallop, and the +94 A/A genotype could be a potential marker available in future selection of Zhikong scallop with heat tolerance.

## Introduction

Zhikong scallop (*Chlamys farreri*) is one of the most important cultured scallop species and contributes greatly to the economic development in the costal region of North China. In the past decades, the industry of scallop aquaculture had been suffering summer mass mortalities seriously [1], and the high temperature was suspected to be the main environmental factor for scallop mortality in summer [1]. The information of the molecule response for the heat stress will be helpful for the better understanding of the heat tolerance mechanism, and provide clues to develop strategy for the control of scallop summer mass mortalities.

The heat tolerance mechanism of an organism was due to the involvement of some molecules such as heat shock proteins (HSPs) [2]. All major classes of HSP are proposed to act as molecular chaperones, helping organisms to modulate stress response and protect organisms from environmentally induced cellular damage [2]–[6]. For example, Hsp101 was confirmed to be essential for thermotolerance by promoting ATP-dependent dissolution of cytosolic or nuclear protein aggregates formed during heat stress [7]. Hsp70s are molecular chaperones involved in a variety of cellular processes, and the thermotolerance of *Drosophila* was positively associated with the expression of Hsp70 [8]. In *Arabidopsis*, the thermotolerance reduced in cytosolic Hsp70-antisense plants [9], while enhanced in cytosolic Hsc70-1 over-expressed plants [10]. Over-expression of hsp22.4 gene in yeast [11] and human Hsp27 gene in rodent cells [12] conferred them thermotolerance. In scallop, even several HSP genes have been identified and they can response to different environmental stresses [13]–[18], the detailed mechanism of their involvement in heat tolerance is still far from well understood.

Recent studies revealed the association between the mutation or sequence polymorphism of HSPs and heat tolerance. For example, the SNP 2437 T/C in the coding region of human *HSPA1L* is associated with the heat shock response by affecting the chaperone function [19]. The SNP -660 C/G in the promoter region of ovine *HSP90AA1* is associated with the adaptation to differential thermal conditions [20]. The allelic variations in *hsp70*, *hsr-omega* and *hsp68* of *Drosophila melanogaster* are associated with natural heritable variation for hardened heat resistance [2], [21], [22]. These accumulating results provided not only the evidences for the study on the mechanism of heat tolerance, but also the candidate markers for the marker assisted selection (MAS) of heat tolerance cultured animals.

HSP22s, a member of small heat shock proteins family with approximately molecular weight of 22 kDa, have been identified in many organisms [7], [23]–[26] for its important role in protecting the cell against heat stress [6], [27], oxidative stress [28], apoptosis [29] and various human diseases [30], [31]. The mutation K141E in human HSP22 is associated with the development of distal motor neuropathy type II [32], and the mutations, K137E and K141E in human HSP22 are associated with Charcot-Marie-Tooth type 2 disease [33]. These mutations are believed to destabilize the structure of HSP22 and decrease its chaperone-like activity in vitro [34], [35]. However, in aquatic animals, despite increasing cognition of structure characteristic and multiple functions of HSP22 [13], [16], [36], there is no report about the association between the polymorphisms of HSP22 and heat tolerance.

In the present study, the polymorphism in the coding region of scallop HSP22 gene from *Chlamys farreri* (CfHSP22) was screened, and its association with the susceptibility/tolerance to high temperature stress was investigated to provide more evidence for the possible mechanism of heat tolerance, and potential markers for selective breeding.

## Materials and Methods

### Ethics statement

The scallops used in the present study are marine cultured animals, and all the experiments are conducted according to the regulations of local and central government.

### Scallops and high temperature treatment

Two hundred Zhikong scallops with approximately 55 mm in shell length were collected from different scallop farms in Qingdao, China, and kept in aerated seawater at 18°C for a week before processing.

For the temperature treatment experiment, scallops were divided into five groups (40 scallops in each group). Four groups were cultivated in 24 L tanks containing aerated seawater at 30°C, and the rest forty scallops were still kept in aerated seawater at 18°C and used as the control group. The seawater in the tanks was changed twice with the same temperature fresh seawater in the first 2 days, and then once up to the end of the experiment. The scallop mortalities in all the groups were recorded every 3 hours from 8:00 to 23:00 until they were sampled at 90 h post treatment. The scallops died in the first one-half period (45 h) of the experiment were classified as susceptible stock and the individuals that survived through the treatment were used as resistant stock. The adductor muscle of each scallop from these two stocks was removed and kept at −80°C until DNA isolation.

About 100 mg adductor muscle from each scallop was homogenized in 500 mL buffer containing 100 mM/L EDTA, 10 mM/L Tris-HCl, pH 8.0, 1% SDS and 0.1 mg/mL Proteinase K (Merck). The genomic DNA was extracted by proteinase K and phenol method as previously described [37].

### Identification and analysis of polymorphisms in the coding region of CfHSP22

A pair of gene specific primers, CfHSP22F and CfHSP22R, was designed based on the sequence of CfHSP22 (AY362760) and used to amplify a 268 bp fragment of the coding region. PCR reaction was performed in a PTC-100 Programmable Thermal Controller Cycler (MJ Research) in 25 µL reaction volume containing 50 ng of DNA template, 1 µL of each primer (10 mM/L), 2.5 µL of 10x PCR buffer, 1.5 µL of MgCl_2_ (25 mM/L), 2 µL of dNTP mix (2.5 mM/L), 15.8 µL of PCR grade water and 0.2 µL (1U) of Taq polymerase (TaKaRa). The PCR temperature profile was as follows: 94°C for 5 min; 35 cycles of 94°C for 30 s, 64°C for 30 s and 72°C for 30 s; a further 10 min elongation at 72°C.

The PCR products from five susceptible scallops and five resistant scallops were detected by electrophoresis on 1% agarose gels. The objective fragments were purified from the gels and cloned into pMD18-T vector (TaKaRa). The constructs were transformed into *E. coli* Top10, and at least two positive clones for each fragment were sequenced by using ABI 3730 Automated Sequencer (Applied Biosystem). The alignments of nucleotide sequence of CfHSP22 coding region were performed by using Vector NTI Suite 9 and the polymorphisms in the coding region of different scallops were analyzed by using Primer Premier 5.0.

### Screening of SNP at locus +94 and the analysis of its association with heat tolerance of scallops

The SNP at locus +94 was screened by Bi-PASA PCR to examine its association with susceptibility of Zhikong scallops to high temperature. Two inner allele-specific primers, A and B, were first designed, with the 3′ base of each primer matching the +94 A-C allele base, and GC tails (GGGGGGGGGC) were added to each of the two allele-specific primers (Table 1). Then two outer primers, P and Q were designed matching the inner allele-specific primers A and B, respectively (Table 1). PCR reaction was performed in a PTC-100 Programmable Thermal Controller Cycler (MJ Research) in 25 µL reaction volume same as described in above section, excepting that two pairs of primers were used. The PCR profile was as follows: initial denaturation at 94°C for 5 min; 30 s at 94°C, 30 s Touch-down from 68°C to 60°C in 0.8°C steps followed by 25 cycles at 60°C, 30 s at 72°C; a final extension at 72°C for 10 min. The Bi-PASA PCR products were detected by electrophoresis on 2% agarose gels, stained with 0.5 µg/mL of ethidium bromide and photographed by Quantity one system (Bio-Rad).

**Table 1:** Primers used in this study.

Primer name	Sequence (5′-3′)	PCR objective
CfHSP22F	GTCGCCATGTACAGGCCGTAGCTC	DNA cloning
CfHSP22R	TTCTTGTCAACATTCAACTCGGCTGCT	DNA cloning
P	GCGGCTCTGTCGCCATGTAC	Bi-PASA PCR
Q	TCTCCCTTGATACACGCCCG	Bi-PASA PCR
A	GGGGGGGGGCGATCCAACATCCCCAAAA	Bi-PASA PCR
B	GGGGGGGGGCTGGTCGGAAGAAGAACTG	Bi-PASA PCR
ReF	ATCGAGCTCATGTATCGCGCAAGAAGTGT	Recombination
ReR	CTGAAGCTTTTTAGAAGATGGCTACCG	Recombination

The genotypes of locus +94 from both stocks were tested for Hardy–Weinberg equilibrium (HWE). SHEsis (http://analysis.bio-x.cn) [38] was used to estimate allele and genotype frequencies and analyze their association with susceptibility/tolerance of scallops to high temperature. A P-value less than 0.05 was accepted as significant.

### Mortality analysis of scallops with different genotypes

Additional 300 scallops were collected to validate the occurrence of different genotypes at locus +94, and the susceptibility/tolerance of scallops with different genotypes to acute thermal stress was also examined by repeating the temperature stress experiment. About 1 µL of haemolymph were collected from each scallop and used as template for Bi-PASA PCR. Each PCR product was detected by electrophoresis on 2% agarose gels, and then stained with 0.5 µg/ml of ethidium bromide to determine the genotypes. Forty scallops with +94 C/C genotype were selected as group I, 40 scallops with +94 A/C genotype were used as group II, 40 scallops with +94 A/A genotype were used as group III, and another 40 scallops (13 individuals with +94 A/A genotype, 13 individuals with +94 A/C genotype and the others with +94 C/C genotype) as control. After being acclimatized in aerated seawater at 18°C for 1 week, scallops in group I, group II and group III were treated by acute thermal stress as described in Section 2.1, while scallops in the control group were still kept in aerated seawater at 18°C during the experiment. The dead scallops were discarded once they were observed and the number were recorded every 12 h. The cumulative mortalities of group I, group II and group III were compared using χ^2^ analysis and the statistical significance was determined by SPSS 11.5 software.

### Recombinant expression of CfHSP22 variants

The cDNA fragments encoding the mature peptide of CfHSP22 with +94 A/A or +94 C/C genotype were amplified with specific primers ReF and ReR (Table 1) from different genotype individuals, respectively. A *Sac* I site was added to the 5′ end of primer ReF and a *Hind* III site was added to the 5′ end of primer ReR after the stop codon. The PCR fragments were cloned into pMD18-T simple vector (TaKaRa), digested completely by restriction enzymes *Sac* I and *Hind* III (NEB), and then cloned into the *Sac* I/*Hind* III sites of expression vector pET-30a (Novagen). The recombinant plasmids (pET-30a-+94 A CfHSP22 and pET-30a-+94 C CfHSP22) were transformed into *E. coli* BL21 (DE3)-pLysS (Novagen). The positive clones were screened by PCR with primers ReF and ReR, and confirmed by further nucleotide sequencing. The positive transformants were incubated in LB medium (containing 50 µg/mL ampicillin) at 37°C with shaking at 220 rpm. When the culture mediums reached OD_600_ of 0.5–0.7, the cells were incubated for 4 additional hours with the induction of IPTG at the final concentration of 1 mM/L. Two His-tagged fusion proteins, 32K rCfHSP22 and 32Q rCfHSP22 (CfHSP22K and CfHSP22Q) were purified by a Ni^2+^ chelating Sepharose column, pooled by elution with 400 mM/L imidazole under denatured condition (8 M/L urea). The purified protein was refolded in gradient urea-TBS glycerol buffer (50 mM/L Tris-HCl, 50 mM/L NaCl, 10% glycerol, 2 mM/L reduced glutathione, 0.2 mM/L oxide glutathione, a gradient urea concentration of 6, 5, 4, 3, 2, 1, 0 M/L urea in each gradient, pH 8.0; each gradient at 4°C for 12 h). Then the resultant proteins were separated by reducing 15% SDS-polyacrylamide gel electrophoresis (SDS-PAGE), and visualized with Coomassie Bright Blue R250. The concentration of purified CfHSP22K and CfHSP22Q was quantified by BCA method [39].

### Examination of molecular chaperone activity of rCfHSP22 variants in vitro

The molecular chaperone activity of CfHSP22K and CfHSP22Q was determined by their ability to protect citrate synthase (CS) against thermal inactivation and help the inactive CS renaturation. In a 0.5 mL microfuge tube, 1 µL CS (pig heart citrate synthase, 8 µg/μL, Sigma) was added to 250 µL Hepes-KOH (100 mM/L, pH 8.0) in the presence or absence of different genotype rCfHSP22 (16 µg). The volume was adjusted to 500 µl with H_2_O and chaperone buffer. As a control, lysozyme (Sigma) was added to a final concentration of 32 µg/mL instead of rCfHSP22. A 20 µL aliquot was removed for the zero time point and CS activity was assayed at room temperature referring to Lee (1995) [40]. The samples were incubated at 38°C for 1 h, and then transferred to 22°C for another 1 h. During the 2 h incubation period, 20 µL aliquots were removed every 10 mins and CS activity was measured. The remaining CS activity was expressed as a percentage relative to the initial activity present at zero time.

## Results

### Scallops susceptible or resistant to high temperature

In the high temperature treatment experiment, the first dead scallop was observed at 12 h after acute thermal stress, and in the following 60 h, the mortality rate of scallops increased gradually and reached the highest level at 72 h. No dead scallop was observed after 84 h, and 87 scallops in total survived after acute thermal treatment until they were sampled at 90 h. The 86 scallops died in the first 48 h were selected as susceptible scallops for their high sensitivity to high temperature, while the 87 scallops survived in the treatment experiment were used as resistant stock, and the rest of the scallops were discarded for their ambiguity. During the whole period, no death occurred in the untreated scallops.

### The polymorphisms in the coding region of CfHSP22

**Figure 1:**
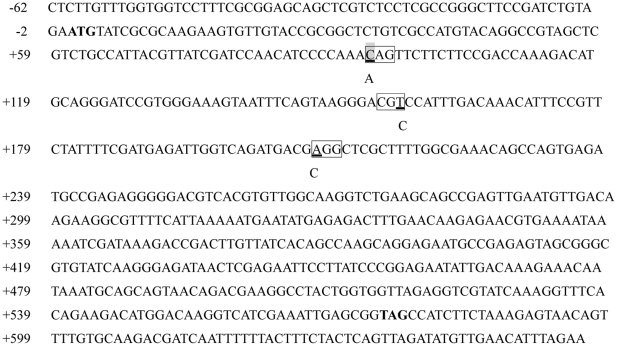
The polymorphisms in the coding region of CfHSP22. The nucleotides are numbered on the left. The polymorphism sites are underlined and the variants are described below. The codons where have mutations are boxed, and the nonsynonymous mutation site is shadowed. The start and stop codons are marked in bold.

A 268 bp fragment of CfHSP22 coding region was amplified from susceptible and resistant scallops, and 15 positive clones from five susceptible scallops and five resistant scallops were sequenced. Three SNPs were found in the amplified coding region, and they were +94 C-A, +157 T-C and +208 A-C (Fig. 1). Among them, only +94 C-A was nonsynonymous mutation, producing an amino acid substitution at codon 32 from a hydrophilic Glutamine to an amphipathic Lysine (Q32K).

### The association between +94 C-A SNP of HSP22 and high temperature susceptibility/tolerance of scallops

**Figure 2:**
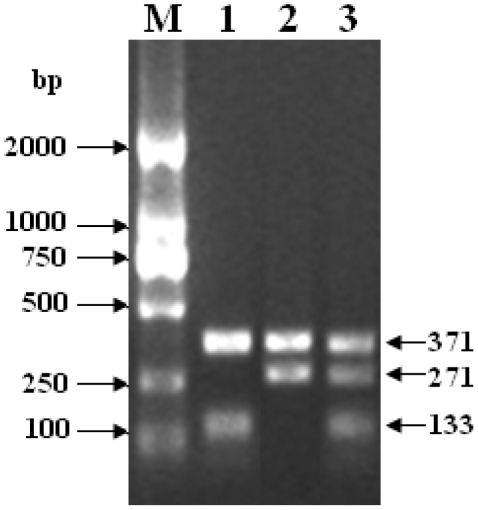
Examination of CfHSP22 +94 locus polymorphism by Bi-PASA PCR. M, DL2000 Marker; 1, +94 C/C genotype; 2, +94 A/A genotype; 3, +94 A/C genotype.

The association between gene polymorphism and high temperature susceptibility/tolerance was investigated by examining the distributions of polymorphism at locus +94 (the +94 C-A SNP) in the susceptible and resistant stocks. For homozygote, the Bi-PASA PCR produced two fragments of 133 and 371 bp in +94 C allele, or two fragments of 271 bp and 371 bp in +94 A allele. While for heterozygote, the Bi-PASA PCR produced three fragments of 133 bp, 271 bp and 371 bp (Fig. 2).

The Hardy–Weinberg equilibrium for genotype frequencies was analyzed with the goodness-of-fit χ^2^-test. Statistical analysis revealed that the genotypic frequency of alleles at locus +94 was not in HWE (*P*<0.01) in both stocks.

At locus +94, the A allele frequency was 18.0% in susceptible stock, while 33.3% in resistant stock. χ^2^-test revealed a significant difference in its frequency distribution between the two stocks (*P*<0.01). There were three genotypes at locus +94, A/A, A/C and C/C, and their frequencies in the resistant stock were 19.5%, 27.6% and 52.9%, while 9.3%, 17.4% and 73.3% in the susceptible stock, respectively (Table 2). There were significant difference between the frequencies of +94 A/A and +94 C/C genotypes in the two stocks (*P*<0.05), as well as between the frequencies of +94 A/C and +94 C/C genotypes (*P*<0.05) (Table 2).

**Table 2:** CfHSP22 +94 genotype distributions in susceptible and resistant stocks.

The SNP +94 A/C		Susceptible stock [No. (%)]	Resistant stock [No. (%)]	χ^2^ (*P*)
Allele	A	31 (18)	58 (33.3)	10.61 (0.001)
	C	141 (82)	116 (66.7)	
Genotype	A/A	8 (9.3)	17 (19.5)	5.43 (0.02)
	A/C	15 (17.4)	24 (27.6)	4.31 (0.038)
	C/C	63 (73.3)	46 (52.9)	
HWE χ^2^ (*P*)		14.43 (0.0001)	12.51 (0.0004)	

**Figure 3:**
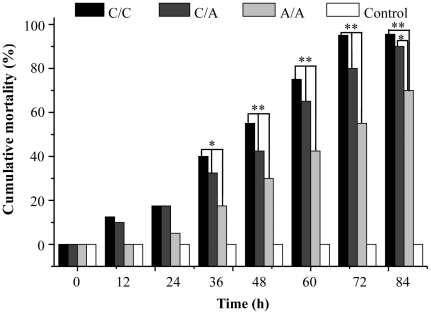
Cumulative mortality of scallops with different genotypes during high temperature treatment. The double-asterisk indicates statistically significant difference of cumulative mortality between scallops with +94 C/C and +94 A/A genotype at 84 h (*P*<0.01). The single-asterisk indicates statistically significant difference of cumulative mortality between scallops with +94 C/C and +94 A/A genotype at 84 h (*P*<0.05). (*: *P*<0.05, **: *P*<0.01).

Another experiment was conducted to further confirm the association between +94 A/A genotype and heat tolerance of scallops. Three hundred scallops were genotyped according to the variation at locus +94, and 40 scallops of each genotype were treated by acute heat stress. The death of group I (+94 C/C genotype) occurred at 8 h after treatment, while the death of group III (+94 A/A genotype) occurred at 20 h after treatment. The semilethal time of scallops with +94 C/C, +94 A/C and +94 A/A genotype was 42 h, 50 h, and 67 h after acute heat treatment, respectively. After 84 h of treatment, only two scallops with +94 C/C genotype and four scallops with +94 A/C genotype survived, while 12 scallops with +94 A/A genotype were still alive. The cumulative mortality between group I and group III (95% and 70%) was significantly different (*P*<0.01). Similarly, there were significant difference in the cumulative mortality between group II and group III (90% and 70%) (*P*<0.05). During the whole period, no scallops died in the control group (Fig. 3).

### Effects of CfHSP22K and CfHSP22Q on the thermal inactivation of CS and its renaturation

**Figure 4:**
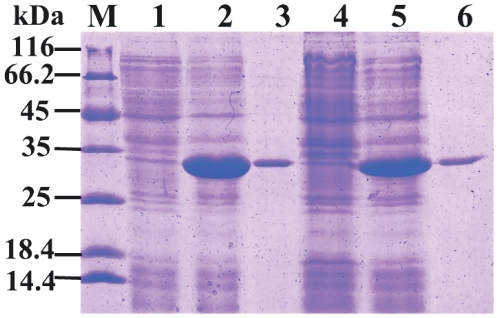
SDS-PAGE analysis of rCfHSP22K and rCfHSP22Q. Lane M: protein molecular standard (kDa); lane 1: negative control for rCfHSP22Q (without induction); lane 2: 1mM IPTG induced rCfHSP22Q; lane 3: purified rCfHSP22Q; lane 4: negative control for rCfHSP22K (without induction); lane 5: 1mM IPTG induced rCfHSP22K; lane 6: purified rCfHSP22K.

**Figure 5:**
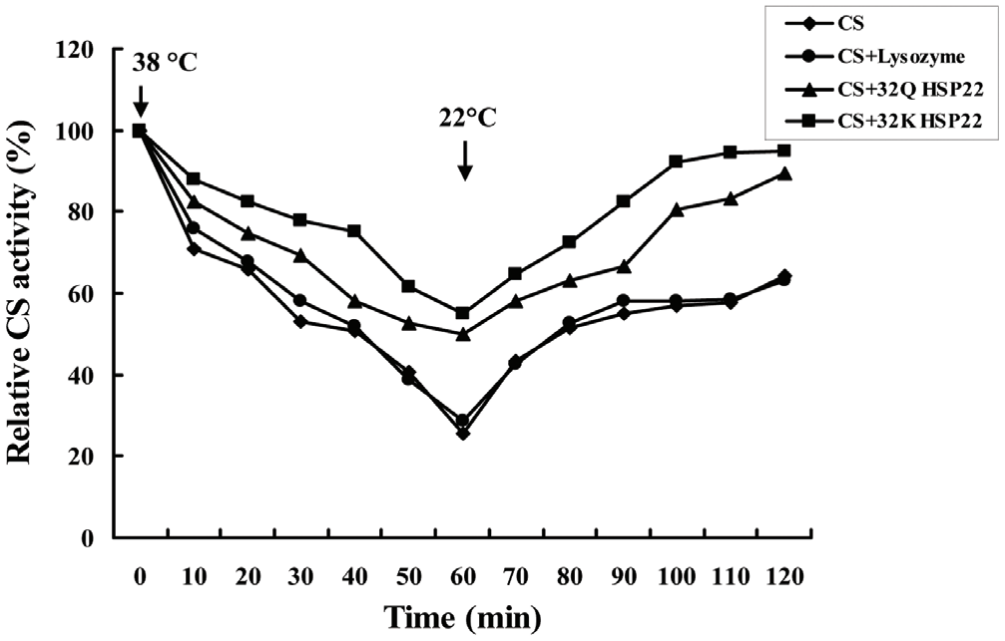
Effects of rCfHSP22K and rCfHSP22Q on the thermal inactivation of CS and its renaturation. CS monomers (16 µg/mL) were incubated at 38°C in the absence (♦) or presence of 32 µg/mL lysozyme (•) or 32 µg/mL rCfHSP22Q (▴) or 32 µg/mL rCfHSP22K (▪). The CS activity was analyzed every 10 mins during the incubation period. Where the arrow indicated, samples were shifted to 22°C.

After IPTG induction, the whole cell lysates of *E. coli* BL21(DE3)-pLysS with pET-30a-+94 A CfHSP22 or pET-30a-+94 C CfHSP22 were analyzed by SDS-PAGE, and distinct bands with molecular weight of ∼28 kDa were revealed, which were consistent with the predicted molecular mass (Fig.4). CS, as a kind of heat-sensitive enzyme, lost 74% activity after the heat treatment at 38°C for 1 h in this study. After recovering for 1 h at low temperature (22°C), partial renaturation of CS could be observed, and CS activity recovered to 64% of the original activity (Fig. 5). When rCfHSP22 was incubated with CS at a ratio of 2∶1 (Hsp22/CS, w/w), rCfHSP22 offered significant protection to CS, while the extent of protection was different for CfHSP22K and CfHSP22Q. After 1 h incubation at 38°C, CS mixed with CfHSP22Q lost 50% activity while CS mixed CfHSP22K lost 45% activity. During the following 1 h restoration at 22°C, CS activity recovered to 89% and 95% of the original activity, respectively (Fig. 5). However, there was no significant difference between the two groups. In the control group, the presence of lysozyme did not affect the inactivation rate (71%) and reactivation rate (63%) of CS (Fig. 5).

## Discussion

The scallop aquaculture in China has been suffering a big challenge because the continuous high temperature seriously affected the production [1]. The strategy for scallop mortality control is urgently needed and selective breeding for high temperature resistant strains is considered as one of the promising solutions. The polymorphisms of certain genes have been identified to be associated with some certain traits, and these polymorphisms could be used as molecular markers to assist selective breeding of strains with certain characteristics. However, to our best knowledge, except that two QTLs influencing heat tolerance have been identified in three rainbow trout half-sib families [41], no other polymorphisms associated with heat tolerance traits have been identified in aquaculture animals.

In the present study, CfHSP22, a small heat shock protein gene previously identified in our laboratory [13], was chosen as the candidate gene for the association analysis. Three SNPs were identified in the coding region of CfHSP22, and only +94 C-A was nonsynonymous mutation. The +94 A/A genotype was more prevalent in resistant stock than that in susceptible one (*P*<0.05), indicating that +94 A/A genotype was associated with the tolerance phenotype of Zhikong scallops to high temperature. In addition, the cumulative mortality of scallops with +94 C/C genotype was significantly higher than that of scallops with +94 A/A genotype (*P*<0.01) after high temperature treatment for 84 h, which further validated the association between +94 A/A genotype and the tolerance of Zhikong scallop to high temperature. Considering the association studies between gene polymorphisms and heat tolerance variation are on the rise [2], [19]-[22], the results in present study throw lights on the mechanism of heat tolerance.

Although the finding of association between HSP22 polymorphisms and some certain traits is of high interest, the detailed mechanisms under the phenomenon are still far from well understood. Inspiringly, accumulating evidences have suggested that genetic polymorphisms in the coding regions could affect proteins activity [42], [43], which could perhaps explain why they were associated with some certain traits. For example, the mutation of Valine to Leucine can change the biological activity of the antimicrobial peptide protegrin-1 [42], the specific mutations in Cosmc from patients with Tn syndrome lead to the loss of its chaperone function [44], and the nonsynonymous mutation in the coding region of human Hsp70 gene can affect the chaperone function [19]. Therefore, it seems reasonable to claim that the change of Glutamine to Lysine in CfHSP22 can have molecular functional significance with respect to the stability and chaperone activity of this protein. In the present study, molecular chaperone activity of rCfHSP22 variants was examined using CS as the model substrate. The rCfHSP22K displayed higher ability to slow down the thermal inactivation of CS (5%), and help inactive CS to recover its activity, which indicated +94 A/A genotype produced HSP22 protein with higher molecular chaperone activity.

It is clear that +94 A-C polymorphism affect the molecular chaperone activity of CfHSP22, and consequently, influence the susceptibility/tolerance phenotype of Zhikong scallops to high temperature, but the molecular mechanism is still unclear. It was unfortunate the change of 32 Q-K was not located in the functional domain of CfHSP22 tertiary structure predicated by SWISSMODEL prediction algorithm (http://swissmodel.expasy.org/) (data not show). The analysis by the Protean program of DNAStar software revealed that 32 Q-K might be involved in the formation of β-strands (data not show). There was one presumption that +94 A-C polymorphism might affect CfHSP22 function through amino-terminal extension, which modulated oligomerization, subunit dynamics and substrate binding. There were reports that the oligomerization and chaperone activity of several sHSPs were influenced by the sequence variations of amino-terminus [45]–[50]. For example, mouse Hsp25 truncated at the amino-terminus produced oligomers of 12-13 subunits, which was larger than the αA-crystallin but lacking chaperone activity [49]. Replacing phenylalanines 24 and 27 within the conserved amino-terminal motif 22-RLFDQFF-28 of murine αB-crystallin with positively charged arginines disrupted the adjacent area and impairs chaperone activity [50].

In summary, the sequence polymorphisms in the coding region of CfHSP22 were identified, and the polymorphism of CfHSP22 +94 C-A was significantly associated with heat tolerance phenotype in Zhikong scallop. In addition, the mutation Gln32Lys possibly improved the *in vivo* heat tolerance phenotype by enhancing the chaperone activity of CfHSP22. The results suggested that +94 A/A would be a potential gene marker associated with enhanced heat tolerance, and could be perhaps applied in future molecular selection program of Zhikong scallop.
